# The Content and Principle of the Rare Ginsenosides Produced from *Gynostemma pentaphyllum* after Heat Treatment

**DOI:** 10.3390/molecules28176415

**Published:** 2023-09-03

**Authors:** Xin-Can Li, Fang-Fang Li, Wen-Jing Pei, Jing Yang, Yu-Long Gu, Xiang-Lan Piao

**Affiliations:** School of Pharmacy, Minzu University of China, Beijing 100081, China; lixincan828@163.com (X.-C.L.);

**Keywords:** *Gynostemma pentaphyllum*, ginsenoside, SEM, TG-DTG, LC-MS

## Abstract

Ginsenoside Rg3, Rk1, and Rg5, rare ginsenosides from *Panax ginseng*, have many pharmacological effects, which have attracted extensive attention. They can be obtained through the heat treatment of *Gynostemma pentaphyllum*. In this study, scanning electron microscopy (SEM) and thermal gravity-differential thermal gravity (TG-DTG) were employed to investigate this process and the content change in ginsenosides was analyzed using liquid chromatography-mass spectrometry (LC-MS). SEM and TG-DTG were used to compare the changes in the ginsenosides before and after treatment. In SEM, the presence of hydrogen bond rearrangement was indicated by the observed deformation of vascular bundles and ducts. The before-and-after changes in the peak patterns and peaks values in TG-DTG indicated that the content of different kinds of compounds produced changes, which all revealed that the formation of new saponins before and after the heat treatment was due to the breakage or rearrangement of chemical bonds. Additionally, the deformation of vascular bundles and vessels indicated the presence of hydrogen bond rearrangement. The glycosidic bond at the 20 positions could be cleaved by ginsenoside Rb3 to form ginsenoside Rd, which, in turn, gave rise to ginsenoside Rg3(S) and Rg3(R). They were further dehydrated to form ginsenoside Rk1 and Rg5. This transformation process occurs in a weak acidic environment provided by *G. pentaphyllum* itself, without the involvement of endogenous enzymes. In addition, the LC-MS analysis results showed that the content of ginsenoside Rb3 decreased from 2.25 mg/g to 1.80 mg/g, while the contents of ginsenoside Rk1 and Rg5 increased from 0.08 and 0.01 mg/g to 3.36 and 3.35 mg/g, respectively. Ginsenoside Rg3(S) and Rg3(R) were almost not detected in *G. pentaphyllum*, and the contents of them increased to 0.035 and 0.23 mg/g after heat treatment. Therefore, the rare ginsenosides Rg3(S), Rg3(R), Rk1, and Rg5 can be obtained from *G. pentaphyllum* via heat treatment.

## 1. Introduction

The rare ginsenosides Rg3(S), Rg3(R), Rk1, and Rg5 have strong biological activities, such as anti-tumor [1,2,3,4], ameliorate myocardial glucose metabolism, and insulin resistance [5,6], suppress platelet mediated thrombus formation [7], alleviate inflammation [8,9,10], and ameliorate allergic airway inflammation and oxidative stress [11]. They are mainly obtained from *Panax ginseng*. However, they were found in a lower abundance in plant extracts. In addition, *P. ginseng* is usually harvested after 4–6 years of cultivation and various abiotic stresses have led to its quality reduction [12]. Therefore, this study focused on *Gynostemma pentaphyllum*, which has the advantages of a shorter growth cycle, lower environmental requirements, higher survival rate, and lower cost, and is also rich in ginsenoside constituents, which can be used as one of the high-quality sources for extracting ginsenosides.

*Gynostemma pentaphyllum* (Thunb.) Makino, commonly known as Jiaogulan in Chinese, belongs to the Cucurbitaceae family and is naturally distributed in China, Japan, Korea, and southeast Asian countries [13]. It is the only plant containing ginsenoside, except Araliaceae [14]. The whole plant is used as medicine in China. *G. pentaphyllum* has a variety of biological activities, such as anti-tumor, lipid-lowering, antioxidant, neuroprotective, and other effects. Therefore, it is widely used in clinical treatment. However, there have been few studies on its ginsenoside. Our previous study showed that 20(S)-ginsenoside Rg3 and 20(R)-ginsenoside Rg3 were isolated from heat-processed *G. pentaphyllum* [15]. These indicate that *G. pentaphyllum* has good potential as a source of ginsenoside extraction and deserves wider attention and research. However, in previous reports, not enough attention has been paid to the ginsenoside-rich properties of *G. pentaphyllum*. Therefore, this study hopes to draw attention to the fact that *G. pentaphyllum* can be used as a good alternative to ginseng, utilizing its advantages in saponin extraction.

In the traditional craft, these rare ginsenosides can be obtained through specific processing methods, including hydrochloric acid degradation [16,17], microbial enzyme degradation [18,19], and metal ion catalytic degradation [20]. However, their drawbacks are also obvious, for example, metal ion catalyzed degradation often uses the heavy metal ion Fe^3+^ [21,22], which is not environmentally friendly. Although the enzymatic hydrolysis reaction is mild, its treatment process is cumbersome and the yield is low. Therefore, researchers often utilize the acid environment of ginseng itself to heat-treat ginseng to prepare rare ginsenosides.

In previous research on *G. pentaphyllum*, we found that there was a dramatic increase in the content of many ginsenosides after heat treatment. Therefore, we hypothesized that the principle of saponin transformation may exist in *G. pentaphyllum*, similar to that in ginseng, and investigated it in this study. We hope to see the principle of the increase in the content of rare ginsenosides in *G. pentaphyllum* after heat treatment and use this principle to extract as many ginsenosides in *G. pentaphyllum* as possible, so as to make greater use of the medicinal value of *G. pentaphyllum* and improve the utilization rate of the resources of *G. pentaphyllum*. Scanning electron microscopy (SEM) was employed to observe the morphological changes in the leaves of *G. pentaphyllum*. A thermal analysis, thermogravimetric (TG), and differential thermogravimetric (DTG) analysis, were used to detect the thermogravimetric changes in *G. pentaphyllum* before and after heat treatment. The contents of the rare ginsenosides Rg3(S), Rg3(R), Rk1, and Rg5 before and after heat treatment were analyzed using ultra-performance liquid chromatography-mass spectrometry (UPLC-MS). The expected results are of great significance for obtaining rare ginsenoside and expanding the utilization range of plant resources.

## 2. Results

### 2.1. Surface Changes in G. pentaphyllum Leaves before and after Heat Processing

**Figure 1:**
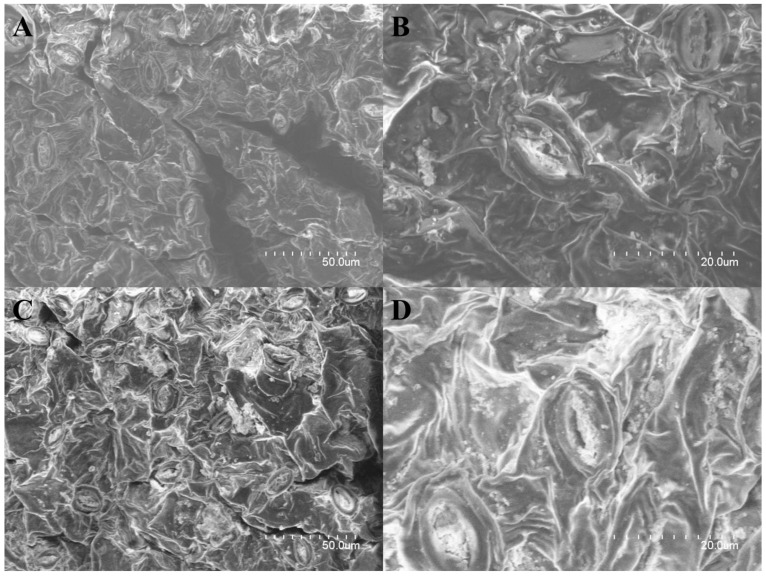
Scanning electron micrographs (SEM) for *G. pentaphyllum* leaves before (**A**,**B**) and after (**C**,**D**) heat treatment.

SEM was used to observe the three-dimensional structures of the leaves’ surfaces visually. The structure of the *G. pentaphyllum* leaves underwent significant changes during the heat processing (Figure 1). The epidermal cells of the raw leaves of *G. pentaphyllum* exhibited a dense, smooth, and full shape. Under high magnification, several verrucous secretions could be observed adhering to the stomata, which were evenly distributed and oval-shaped within the leaves. However, the leaves of the heat-processed *G. pentaphyllum* exhibited noticeable water loss and shrinkage, resulting in a rough and uneven leaf surface. The cellular distribution appeared disordered, making it difficult to discern clear cell boundaries and gaps. The original smooth vessel and vascular bundle structures were significantly deformed due to the high temperature and pressure, leading to plant fiber breakage and numerous irregular shrinkage and tissue faults. The secretion near the stomata increased, which was observed under high magnification, and the stomata embedded in the leaf surface protruded due to tissue structure damage.

The changes observed in the *G. pentaphyllum* leaves following heat processing may be attributed, to a certain extent, to the alteration in the microstructure and bonding relationships among the leaf cells. Firstly, there was an increase in secretion around the stomata. Heat treatment induced alterations in the physicochemical state of the biofilm, resulting in enhanced cell permeability. The rupture of the central vacuole led to the extrusion of many ions and the disruption of the cell wall. The disrupted cell permeability during heat treatment caused the leakage of inorganic salts into the extracellular environment, leading to the formation of inorganic salt crystals on the cell surface. Secondly, there was severe irreversible shrinkage with tissue breaks in the ducts and vascular bundles. The main constituents of conduits and vascular bundles are cellulose, hemicellulose, and lignin, all of which are biomolecules with high molecular weights that bond and interact primarily via hydrogen bonding to maintain the relative stability of the plant form. The pronounced and irreversible deformation of the fibrous structures in the leaves after heat processing may be attributed to the weakened hydrogen bonding between the hydroxyl groups and water molecules in the molecular chains of cellulose. These hydrogen bonds were disrupted by the continuous increase in temperature. Consequently, the free hydroxyl groups formed after the fracture could recombine, generating new intra- and intermolecular hydrogen bonds. This disorder in the arrangement of the molecular chains of cellulose manifested as fiber shrinkage and the breaking of hydrogen bonds also gave the possibility of ginsenoside conversion. Therefore, we hypothesize that, in heat-treated *G. pentaphyllum*, ginsenosides of a larger molecular weight may also break glycosidic bonds to form ginsenosides of a smaller molecular weight. Thirdly, the color of the leaves changed from green to brown. The high temperature led to an increase in the levels of reactive oxygen species (ROS), leading to chlorophyll degradation. This alteration in the ratio of chlorophyll-to-carotenoid in the leaves resulted in the yellowing of the leaves. It was reported that 4-year-old fresh ginseng was steamed at 110 °C for 2 h, followed by drying for 12 h. This process was repeated nine times. The accumulation of Maillard reaction products led to the browning of the ginseng, resulting in black ginseng [23,24,25,26]. Therefore, the change in leaf color may also be attributed to the Maillard reaction occurring in *G. pentaphyllum* leaves during heat treatment.

The high temperature and pressure disrupted the cell wall structure, degraded the hemicellulose, and released the protein enclosed. The released protein molecules then underwent covalent binding with carbohydrates, leading to the Maillard reaction and the production of dark-brown- or black-like macromolecular substances. However, this speculation requires confirmation by determining the content changes in the key indicator components (reducing sugars, amino nitrogen, melanoid, moisture, and polyphenols) involved in the Maillard reaction during the heat treatment process.

### 2.2. Changes in the Internal Structure of G. pentaphyllum before and after Heat Processing

**Figure 2:**
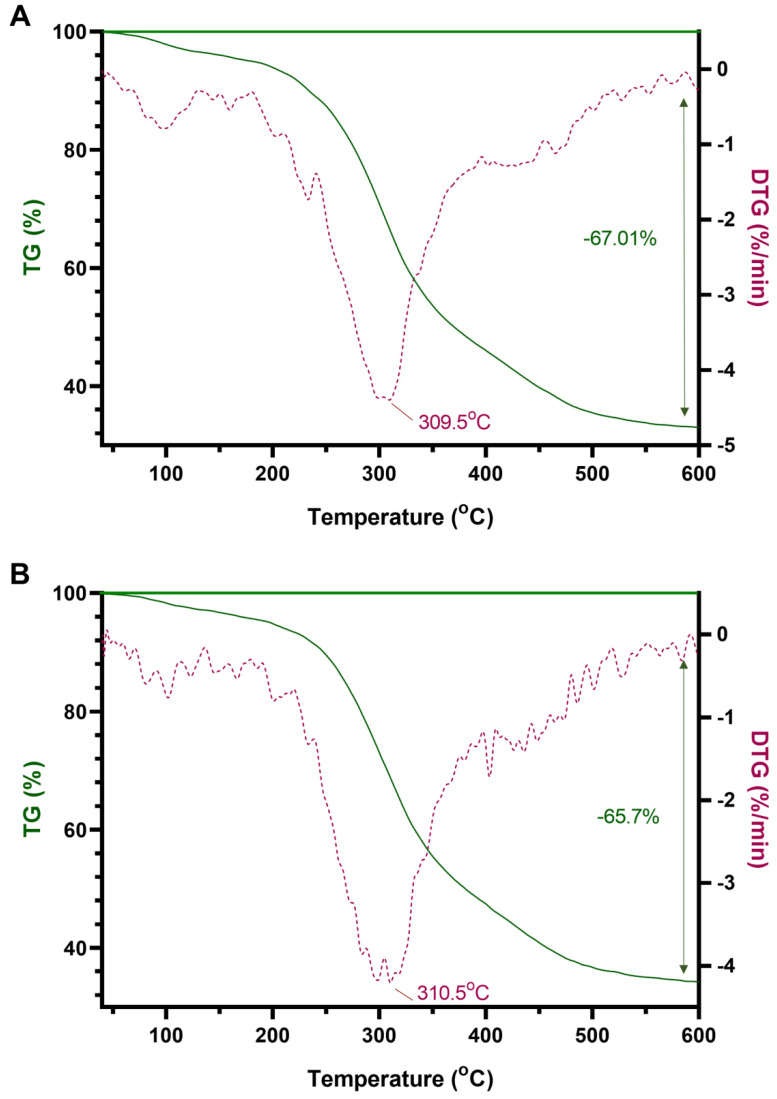
TG-DTG curves of leaves of *G. pentaphyllum* before (**A**) and after (**B**) heat treatment.

The results of the TG analysis showed that the thermal decomposition of *G. pentaphyllum* can be divided into three stages: water loss, rapid weight loss, and slow weight loss. During the first stage, the gradual evaporation of water occurred as the *G. pentaphyllum* powder dried completely, resulting in a slight reduction in quality. In the second stage, chemical bonds in different tissues were sequentially broken in a specific order. For example, proteins broke down to form polypeptides, glycosidic bonds in polysaccharides broke to form monosaccharides, and a large amount of volatiles were simultaneously generated. The third stage involved the gradual pyrolysis of the sample, with the sample quality stabilizing after reaching 600 °C (Figure 2). A comparison of the weight loss ratio before and after the heat treatment showed that the heat-processed *G. pentaphyllum* had less weight loss, demonstrating that the internal structure changed and the generation of new high-temperature-resistant substances that are not easily decomposed. For instance, cellulose rearranged through hydrogen bonds, increasing the proportion of hydrogen bonds in the molecule and enhancing its stability.

From the DTG curves, it is evident that the heat treatment induced variations in the types and contents of the nitrogen-containing compounds, glycosides, acids, esters, and other substances from *G. pentaphyllum*. These changes led to alterations in the bonding relationships and proportions of chemical bonds manifested in the different temperatures, peak heights, and number of extreme values and characteristic peaks observed in the DTG curves (Figure 2). This evidence confirmed that the heat treatment affected the bonding status of the original chemical bonds in the ginsenosides, such as the formation of ginsenoside Rg3(S), Rg3(R), Rk1, and Rg5, resulting from the breakage of one glycosidic bond by ginsenoside Rd or Rb3 and leading to a decrease in the glycosidic bond characteristic peaks.

### 2.3. Production of Rare Ginsenosides from G. pentaphyllum

**Figure 3:**
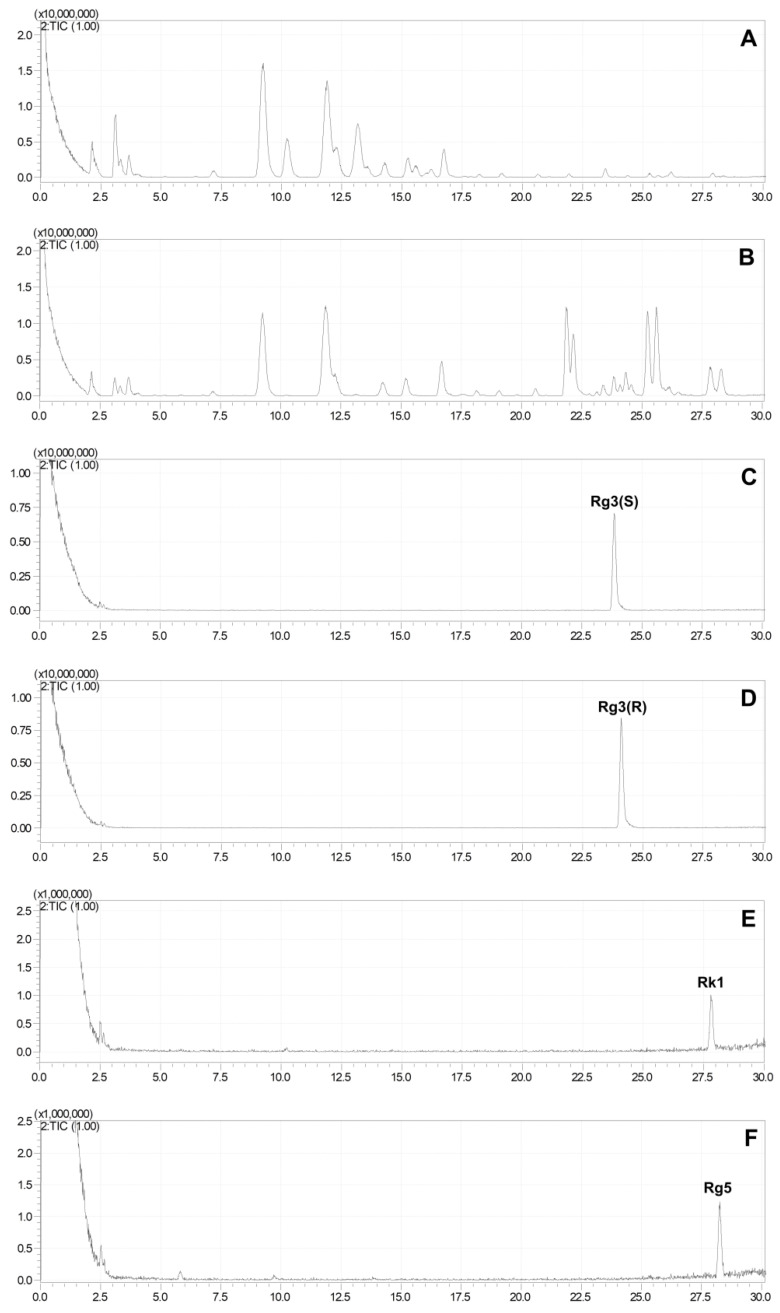
Total ion chromatograms of *G. pentaphyllum*. Extract of *G. pentaphyllum* before (**A**) and after (**B**) heat treatment. Reference standards ginsenoside Rg3(S) (**C**), Rg3(R) (**D**), Rk1 (**E**), and Rg5 (**F**).

The HPLC-MS method was used to compare the differences in the total extracts of *G. pentaphyllum* before and after heat processing, and the results demonstrated significant changes in the chemical components after heat treatment. The polar saponins of *G. pentaphyllum* (8–17 min) decreased, while the low polar saponins (22–30 min) exhibited a substantial increase in both content and species following heat treatment (Figure 3). This indicates that heat treatment can induce effective structural transformations in the chemical composition of *G. pentaphyllum*. By comparing them with reference standards, it was found that the contents of rare ginsenosides, such as ginsenoside Rg3, ginsenoside Rk1, and ginsenoside Rg5, were altered.

**Figure 4:**
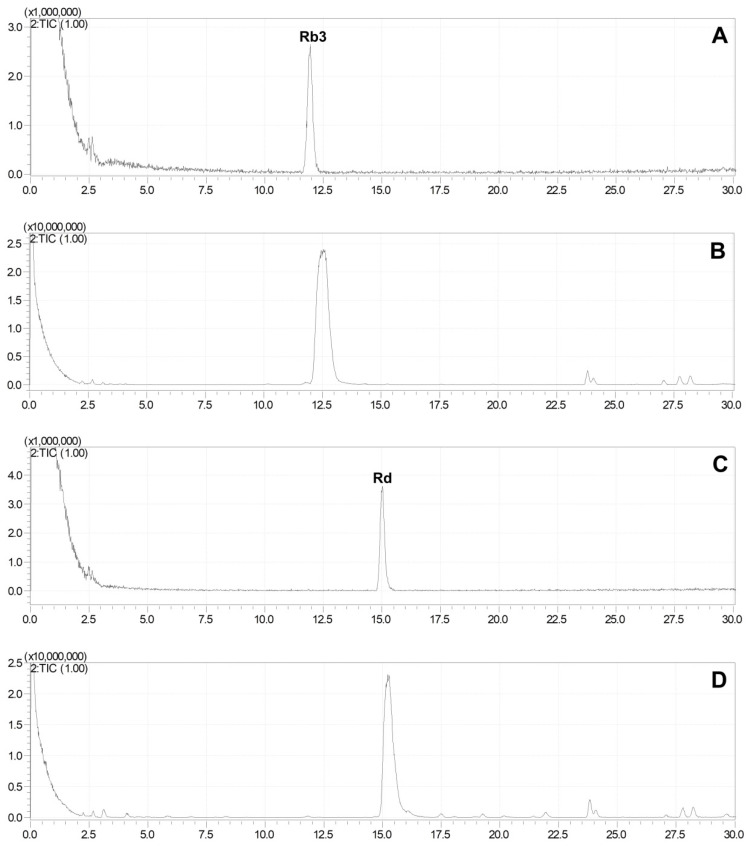
Total ion chromatogram of ginsenoside Rb3 and Rd before and after heat treatment. (**A**) 100 μg/mL of ginsenoside Rb3 solution was injected 1 μL to LC-MS. (**B**) 100 μg/mL of Rb3 solution was steamed for 3 h at 120 °C, 0.24 MPa and injected 1 μL to LC-MS. (**C**) 100 μg/mL of ginsenoside Rd solution was injected 1 μL to LC-MS. (**D**) 100 μg/mL of Rd solution was steamed for 3 h at 120 °C, 0.24 MPa and injected 1 μL to LC-MS.

**Figure 5:**
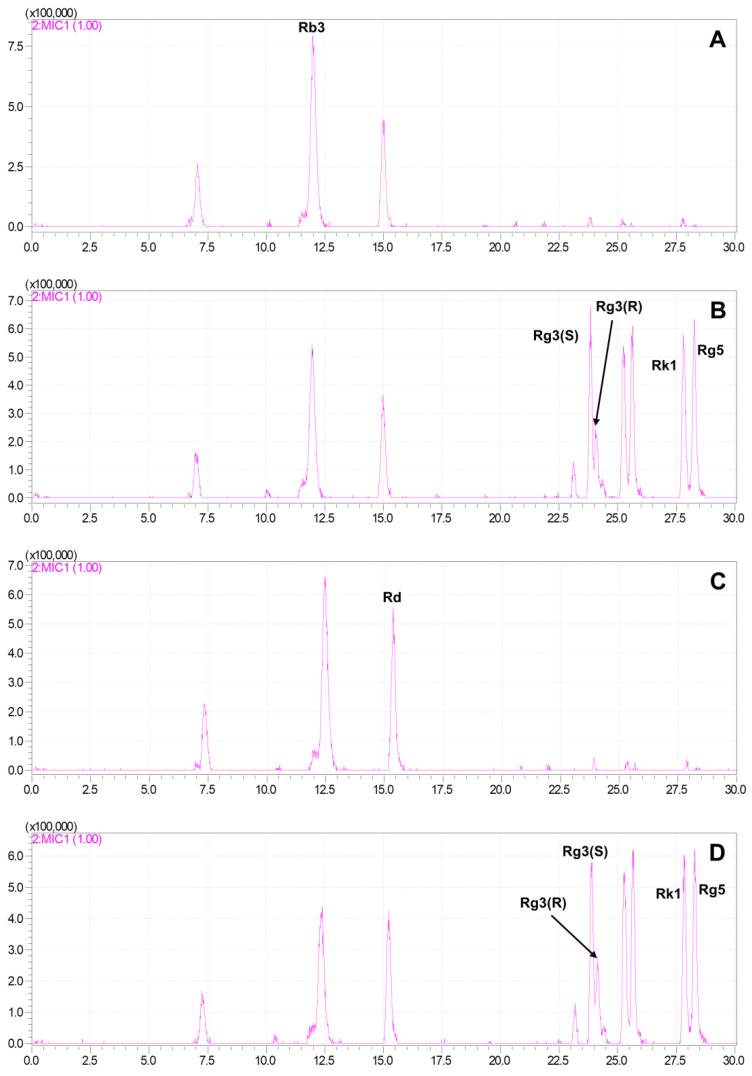
Multi ion chromatogram (MIC) of *G. pentaphyllum* mixed with polar ginsenosides. In total, 100 mg of *G. pentaphyllum* was mixed with 500 μL of 100 μg/mL of ginsenoside Rb3 (**A**) or 100 μg/mL of Rd (**C**) and extracted with 80% methanol to a volume of 20 mL. In total, 100 mg of *G. pentaphyllum* was mixed with 500 μL of 100 μg/mL of Rb3 (**B**) or 100 μg/mL of Rd (**D**) and steamed for 3 h at 120 °C, 0.24 MPa, and extracted with 80% methanol to a volume of 20 mL. The extracts were filtered through 0.22 μm microporous membranes.

To eliminate the interference of other compounds, 500 μL of ginsenosides Rb3 (100 μg/mL) and 200 μL of Rd (100 μg/mL) aqueous solutions were separately added to 100 mg of *G. pentaphyllum*, followed by heat treatment. The results indicated that the ginsenosides Rb3 and Rd in the water almost remained unchanged after the heat treatment (Figure 4), while their degradation was observed clearly in *G. pentaphyllum*, and the amount of ginsenoside 20(S)-ginsenoside Rg3, 20(R)-ginsenoside Rg3, Rk1, and Rg5 increased (Figure 5).

**Figure 6:**
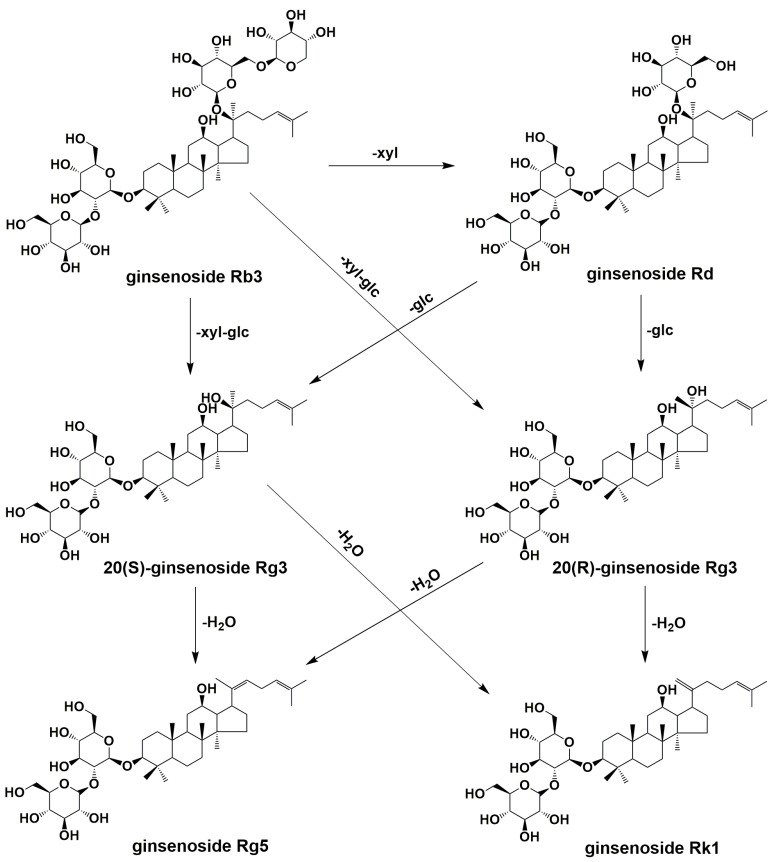
Proposed pathway of ginsenosides of *G. pentaphyllum* after heat treatment.

Ginsenoside Rb3 generated a small amount of ginsenoside Rd and significant amounts of ginsenoside Rg3(S), Rg3(R), Rk1, and Rg5, while ginsenoside Rd primarily yielded ginsenoside Rg3(S), Rg3(R), Rk1, and Rg5. These findings suggest the existence of a transformation relationship between ginsenoside Rb3, Rd, Rg3(S), Rg3(R), Rk1, and Rg5 (Figure 6). However, this transformation can only occur in a specific environment created by one or more substances within *G. pentaphyllum* and cannot solely be attributed to hydrolysis caused by a high temperature and high pressure.

In order to observe the chemical environment of *G. pentaphyllum* before and after heat treatment, the pH of its extracts was detected. The extract of *G. pentaphyllum* had a weak acidic pH of approximately 6, which decreased to about 5 after the heat treatment. Both pH values fulfilled the requirement for hydrolysis and the cleavage of glycosidic bonds under acidic conditions. This result indicated that the glycosidic bond of saponins can be cleaved through dilute acid with free hydrogen ions. Therefore, the weak acidity of the *G. pentaphyllum* extract was the underlying cause of saponin decomposition.

### 2.4. Quantitative Analysis of Ginsenosides of G. pentaphyllum before and after Heat Processing

Each ginsenoside solution was injected directly into a mass spectrometer by a syringe pump at 5 μL/min to optimize the experimental parameters automatically and manually. The negative ion mode was chosen for the ginsenosides due to the higher signal intensity of [M-H + HCOOH]^−^ and [M-H]^−^ ions. The pairs of quantitative ions in MRM mode were selected and their collision energies were obtained. UPLC was coupled to a mass spectrometer after direct MS optimization.

**Figure 7:**
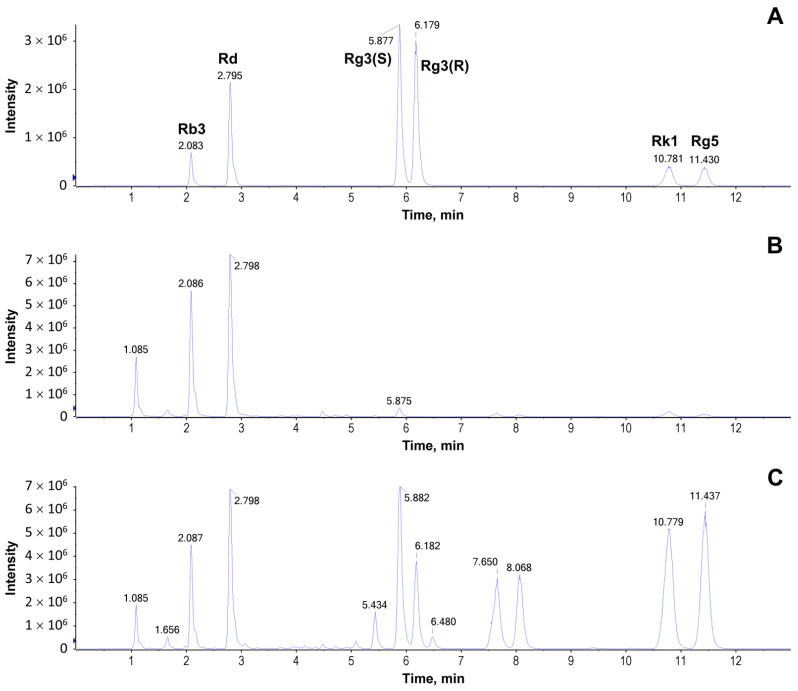
LC-MRM-MS of the extract of *G. pentaphyllum*. (**A**) Mixed standard reference solution. (**B**) Extract of *G. pentaphyllum*. (**C**) Extract of heat-processed *G. pentaphyllum*.

The quantitative analysis of Rb3, Rd, Rg3(S), Rg3(R), Rk1, and Rg5 from GP and HGP was carried out using the UPLC-MRM-MS method. They were effectively separated within 13 min using a Waters Acquity UPLC BEH C18 column (2.1 × 50 mm, 1.7 μm) (Figure 7). Their linearity was investigated by calculating the correlation coefficients (r) of regression curves. The regression equation and linear range of ginsenosides Rb3, Rd, Rg3(S), Rg3(R), Rk1, and Rg5 were obtained, which all showed a good linear relationship. The contents of the six ginsenosides in *G. pentaphyllum* before and after heat processing are presented in Table 1. The results revealed that the content of ginsenoside Rb2 from *G. pentaphyllum* degraded after heat processing, while the contents of ginsenoside Rd, 20(S)-ginsenoside Rg3, 20(R)-ginsenoside Rg3, ginsenoside Rk1, and ginsenoside Rg5 increased, especially the contents of 20(S)-ginsenoside Rg3, 20(R)-ginsenoside Rg3, ginsenoside Rk1, and ginsenoside Rg5, which increased significantly.

## 3. Discussion

In this study, we found a significant increase in and enrichment of the rare ginsenosides Rg3(S), Rg3(R), Rk1, and Rg5 in heat-treated *G. pentaphyllum*.

We hypothesize that this transformation is related to the breaking of chemical bonds, in which the breaking of glycosidic and hydrogen bonds plays a very important role. The ginsenosides Rb3 and Rd may form Rg3(S) and Rg3(R), respectively, through glycosidic bond breaking, and C-20 dehydration occurs in Rg3(S) and Rg3(R) to form Rg5 and Rk1, respectively. High temperatures provide energy for chemical bond breaking, and *G. pentaphyllum*’s own weak acidity provides abundant free hydrogen ions. This reaction can only take place under acidic conditions, which we hypothesize is due to the acetal structure of the glycosidic bond, which is rarely broken under neutral and alkaline conditions.

Heat treatment is common in the processing of ginseng and is often accompanied by changes in ginsenoside content before and after heat treatment. Nam et al. [27] reported a decrease in the contents of ginsenosides Rb1, Rb2, Rc, Rd, Re, Rf, and Rg1, and an increase in the contents of Rg3(S), Rg3(R), and Rk1 in steamed ginseng. The work of Ji et al. [28] also showed that the contents of ginsenosides F4, Rh4, Rg3(S), Rg3(R), Rk1, and Rg5 in ginseng heated for one to two hours were increased in proportion to the duration of the heat treatment. Similar content changes were also observed in *G. pentaphyllum* after heat treatment: the content of ginsenoside Rb3 decreased from 2.25 mg/g to 1.80 mg/g, while that of ginsenosides Rk1 and Rg5 increased from 0.08 and 0.01 mg/g to 3.36 and 3.35 mg/g, respectively. The ginsenosides Rg3(S) and Rg3(R) were almost undetectable in *G. pentaphyllum*, while their contents were almost undetectable in *G. pentaphyllum*, and these contents increased to 0.035 mg/g and 0.23 mg/g after heat treatment, respectively. Wang et al. [29] previously determined the contents of various ginsenosides in *G. pentaphyllum*. Compared to Wang’s results, the contents of ginsenosides Rb3, Rk1, and Rg5 in *G. pentaphyllum* after heat treatment were significantly higher than those of the corresponding ginsenosides in ginseng. Meanwhile, the content of Rg3(R) in heat-treated *G. pentaphyllum* was significantly higher than that in ginseng and close to that in red ginseng.

Although the principle and processing method of heat treatment have been systematically studied and applied in ginseng, the application in *G. pentaphyllum*, which is also rich in ginsenosides, is not yet common. Although some research groups have previously heat-treated *G. pentaphyllum*, they lacked in-depth studies on the effects of heat treatment and the principle of secondary rare saponin production. This study complements and extends the excellent work of Duan et al. [30] by providing a theoretical basis for the application of heat treatment to *G. pentaphyllum* and clarifying that a similar secondary ginsenoside generation principle does exist in *G. pentaphyllum*, expanding the scope of the application of this principle. At the same time, we quantified the ginsenoside content in six ginsenosides before and after heat treatment. The excellent ginsenoside content of heat-treated *G. pentaphyllum* suggests to us that it deserves extensive attention from researchers as a superior source of secondary rare ginsenosides. In particular, the contents of ginsenosides Rb3, Rk1, and Rg5 are even better than those of red ginseng, while the preparation is also more convenient, time-saving, and efficient than that of red ginseng.

We need to conduct more work in the future, and an important goal is to try to find the optimal temperature and reaction time for the heating of ginsenoside conversion via orthogonal experiments, so that the reaction products can be under human control. For example, work can be carried out in the direction of the ginsenoside Rg3(S) and Rg3(R), or the ginsenoside RK1 and Rg5, and thus directed to enrichment of the target ginsenosides.

## 4. Materials and Methods

### 4.1. Chemicals and Materials

*G. pentaphyllum* was purchased in Zhangzhou (Fujian, China) and was professionally identified. The voucher specimen (No. GP 2016-01) was placed in the Isolation and Structure Identification Laboratory in School of Pharmacy, Minzu University of China. The SEM analysis was conducted using a Hitachi S-4800 Cold Field Emission Scanning Electron Microscope (Hitachi, Tokyo, Japan). The TG and DTG analyses were detected using an STA 449f5 Jupiter synchronous thermal analyzer (Netzsch, Free State of Bavaria, Germany). The qualitative HPLC-MS analysis was performed using a LC-20A series Shimadzu HPLC system coupled to an ion trap time-of-flight mass spectrometer (LCMS-IT-TOF) (Shimadzu Co., Ltd., Tokyo, Japan). The UPLC-MRM-MS analysis was performed using an AExionLC™ AD series UPLC system 1.0 (AB Sciex) coupled to a QTRAP 5500 mass spectrometer (AB Sciex, Framingham, MA, USA). The entire system, including the data acquisition and processing, was controlled using AB Sciex Analyst Software Package Version 1.6.3. HPLC-grade acetonitrile and formic acid were purchased from Fisher Chemical Co., Ltd. (Marshalltown, IA, USA). Ultrapure water was obtained from Jilin Wahaha Group Co., Ltd. (Jilin, China), and methanol and ethanol were purchased from Beijing Tongguang Fine Chemicals Co., Ltd. (Beijing, China). Glutaraldehyde fixative was procured from Wuhan Servicebio Technology Co., Ltd. (Wuhan, China). The standard reference compounds ginsenoside Rb2, ginsenoside Rd, 20(S)-ginsenoside Rg3, 20(R)-ginsenoside Rg3, ginsenoside Rg5, and ginsenoside Rk1 were purchased from RENI Pharmaceutical Technology Co., Ltd. (Sichuan, China) with a purity exceeding 98%.

### 4.2. Heat Processing of G. pentaphyllum

The leaves of *G. pentaphyllum* were heat processed for 3 h by steaming at 120 °C and 0.24 MPa using an LDZM-60KCS vertical pressure vapor sterilizer (Shanghai, China) and dried to obtain heat-processed *G. pentaphyllum*.

### 4.3. SEM Analysis

SEM was performed to examine the leaf tissues of *G. pentaphyllum*. Small sections of the leaves, measuring 2 mm × 2 mm, were selected from a consistent location near the middle vein. The samples (1 g) were fixed with 2.5% glutaraldehyde in a phosphoric acid buffer (pH = 7.4, 5 mL) for 12 h. After fixation, the samples were rinsed three times with 0.1 M phosphate buffer (pH 7.4) for 10 min each. Subsequently, a stepwise dehydration process was carried out using ethanol at concentrations of 30%, 50%, 70%, 90%, 95%, and 100% (10 mL/per step and 20 min per step) and a temperature of 4 °C. This gradual dehydration procedure effectively eliminated moisture from the leaf tissues. The dried leaves were then placed in an oven at 65 °C for 30 min until their complete desiccation. Finally, the prepared samples were analyzed using a Hitachi S-4800 Cold Field Emission Scanning Electron Microscope.

### 4.4. TG and DTG Analysis

The pulverized leaves of *G. pentaphyllum*, both before and after the heat processing, were sieved through a 200-mesh screen (approximately 0.075 mm particle size). The obtained samples (1 g each) were naturally dried at 25 °C and then individually placed in crucibles (3 mg for each sample). High-purity nitrogen gas (99.999%) was used as the purging gas at a flow rate of 100 mL/min. The samples were subjected to a heating process under nitrogen protection, starting from 40 °C and gradually increasing to 650 °C at a heating rate of 10 °C/min. The thermogravimetric (TG) and differential thermogravimetric (DTG) curves were recorded using a STA 449f5 Jupiter synchronous thermal analyzer (Netzsch, Germany).

### 4.5. Sample Preparation for HPLC and LC-MS

Approximately 100 mg of the leaves of *G. pentaphyllum,* before and after heat processing, was weighed into a separate 50 mL centrifuge tubes and then about 20 mL of 80% methanol aqueous solutions was added, respectively, to prepare 5 mg/mL of samples. They were extracted for 30 min using a KQ-250E sonicator (KunShai, China). The extracts were filtered through 0.22 μm microporous membranes.

### 4.6. Preparation of Standard Stock Solutions

The standard reference compounds ginsenoside Rb3, ginsenoside Rd, 20(S)-ginsenoside Rg3, 20(R)-ginsenoside Rg3, ginsenoside Rk1, and ginsenoside Rg5 were accurately weighed and dissolved with methanol to obtain 1 mg/mL of ginsenoside Rb3, ginsenoside Rd, 20(S)-ginsenoside Rg3, 20(R)-ginsenoside Rg3, ginsenoside Rk1, and ginsenoside Rg5, respectively. A standard stock solution was prepared by mixing 100 μL of each of the above 6 standard solutions with 400 μL of methanol, which consisted of 100 μg/mL of each standard. It was stored in brown vials at 4 °C and filtered through 0.22 μm membrane filters before analysis.

### 4.7. Chromatography and Mass Spectrometry Analysis

The analysis of the saponins in *G. pentaphyllum* was conducted within 30 min using a ZORBAX SB-C18 column (4.6 × 250 mm, 5 μm). The mobile phase consisted of a gradient elution system using 0.1% formic acid aqueous (A) and 0.1% formic acid acetonitrile (B). The gradient elution conditions were as follows: 0 min, 32% B; 10 min, 35%B; 17 min, 42%B; 30 min, 75%B, with a flow rate of 1.0 mL/min.

For the quantitative analysis, an AExionLC™ AD series UPLC system (AB Sciex) was utilized for chromatographic separation. An Acquity UPLC BEH C18 column (2.1 × 50 mm, 1.7 μm) was employed for the separation process. The mobile phase consisted of 0.1% formic acid aqueous (A) and acetonitrile with 0.1% formic acid (B). The gradient elution was programmed as follows: 0 min, 32% B; 3 min, 36% B; 3.1 min, 40% B; 13 min, 48% B. The flow rate was set at 0.4 mL/min. The sample injection volume was 1 μL. The column temperature was conditioned at 30 °C.

A SCIEX QTRAP 5500 mass spectrometer (AB SCIEX, USA), equipped with an electrospray ionization source (ESI), was used for the quantitative analysis in negative mode and multiple reaction monitoring (MRM) modes. The operation parameters in MRM were set as follows: an ion spray voltage (IS) of −4500 V; turbo spray temperature (TEM) of 550 °C; and ion source gas 1 (GAS1), ion source gas 2 (GAS2), and curtain gas (CUR) were set at 55, 55, and 35 psi, respectively. The declustering potential (DP) and collision energy (CE) were set to match the MRM of each analyte (Table 2). Data acquisition and processing were performed using Analyst Version 1.6.3.

### 4.8. Statistical Analysis

All the quantitative data were expressed as means ± SD. All the statistical analyses were performed using one-way ANOVA by GraphPad 8 (GraphPad Software, San Diego, CA, USA). Statistical significance was defined as *p* < 0.05.

## 5. Conclusions

In conclusion, high-polarity saponins can be converted into low-polarity saponins through heat treatment. This phenomenon is commonly observed in gypenosides and ginsenosides, leading to a substantial increase in the content of rare saponins. The reaction occurs within a weak acidic environment without the need for external catalysts, microorganisms, or enzymes. *G. pentaphyllum* is abundant in organic and acidic amino acids, providing an appropriate pH environment for this reaction. During heat treatment, macromolecular proteins undergo decomposition into small molecules of acidic amino acids, further promoting the degradation of polar saponins.

## Figures and Tables

**Table 1:** Contents of ginsenosides of *G. pentaphyllum* before and after heat processing.

Compound	Content (mg/g)	
	Raw *G. pentaphyllum*	Heat-Processed *G. pentaphyllum*
Ginsenoside Rb3	2.248 ± 0.036	1.798 ± 0.036
Ginsenoside Rd	0.918 ± 0.030	0.917 ± 0.020
20(S)-ginsenoside Rg3	N.D. ^1^	0.035 ± 0.049
20(R)-ginsenoside Rg3	N.D. ^1^	0.231 ± 0.094
Ginsenoside Rk1	0.083 ± 0.009	3.359 ± 0.463
Ginsenoside Rg5	0.006 ± 0.004	3.347 ± 0.410

^1^ Note: N.D., not detected.

**Table 2:** Quantitative ion pairs of ginsenosides and optimized parameters.

Compound	Retention Time (min)	Quantitative Ion Pair (m/z)	DP (V)	CE (V)	CXP (V)
Ginsenoside Rb3	2.08	1123.6/1077.6	−40	−34	−21
Ginsenoside Rd	2.80	991.4/945.5	−25	−38	−25
20(S)-ginsenoside Rg3	5.88	829.6/621.4	−40	−54	−27
20^®^-ginsenoside Rg3	6.18	829.5/783.5	−60	−25	−13
Ginsenoside Rk1	10.78	811.5/765.5	−55	−30	−15
Ginsenoside Rg5	11.43	811.6/765.5	−55	−30	−15

## Data Availability

Not applicable.
